# Effect Observation of Electro-Acupuncture Anesthesia Combined with General Anesthesia in Elderly Patients Undergoing Gastrointestinal Tumor Resection

**DOI:** 10.3389/fsurg.2022.901638

**Published:** 2022-05-12

**Authors:** Jiping Xu, Peng Li, Liyan Zheng, Qiong Chen

**Affiliations:** ^1^ Department of Anesthesiology, Rizhao Central Hospital, Rizhao City, China; ^2^ Department of Anesthesiology, Yongkang First People's Hospital, Jinhua City, China

**Keywords:** anesthesia, acupuncture, gastrointestinal tumor resection, inflammatory factors, stress state, T lymphocyte subsets

## Abstract

**Objective:**

To investigate the anesthetic effect of electro-acupuncture (EA) anesthesia combined with general anesthesia in elderly patients undergoing gastrointestinal tumor resection, and to analyze the effects of EA anesthesia on inflammatory factors, stress state and T lymphocyte subsets in elderly patients.

**Methods:**

Total of 118 elderly patients who underwent gastrointestinal tumor resection in our hospital from June 2018 to March 2021 were selected and divided into the control group (59 cases) and the observation group (59 cases) according to the random number method. General anesthesia was adopted in the control group and EA anesthesia combined with general anesthesia was adopted in the observation group. The anesthesia effect, stress state, levels of inflammatory factors, T-lymphocyte subsets and adverse reactions were compared.

**Results:**

The VAS score, agitation score and respiratory normalization time in the observation group were lower than those in the control group (*p* < 0.05). After surgery, the levels of serum Cor, ET, NE and DA in the observation group were lower than those in the control group (*p* < 0.05). At 24 h after surgery, the levels of serum TNF-α, IL-6 and IL-1β in the observation group were lower than those in the control group (*p* < 0.05). At 24 h after surgery, the levels of $CD_{3}{}^{+}$, $CD_{4}{}^{+}$, and $CD_{4}{}^{+}//CD_{8}{}^{+}$ in the two groups were lower than those before surgery, and the levels of $CD_{3}{}^{+}$, $CD_{4}{}^{+}$, and $CD_{4}{}^{+}//CD_{8}{}^{+}$ in the observation group were higher than those in the control group (*p* < 0.05). During the hospitalization, the total incidence rate of adverse reactions after anesthesia in the observation group was lower than that in the control group (*p* < 0.05).

**Conclusion:**

EA anesthesia combined with general anesthesia has good anesthesia effect when used for gastrointestinal tumor resection in the elderly. It can stabilize the internal environment of patients, alleviate postoperative stress response and inflammatory response, and regulate the body immune function. Moreover, it has high safety and can significantly reduce the occurrence of postoperative adverse reactions.

## Introduction

Resection of gastrointestinal tumors in elderly patients is a common treatment option in abdominal surgery and is performed under general anesthesia. Traumatic surgery often leads to stress state in patients and stimulates the immune system to release a large number of pro-inflammatory factors, damage vascular endothelial cells, and inhibit normal immune function (1, 2). Elderly patients are more likely to suffer from hemodynamic disorders, aggravate surgical stress trauma, cognitive impairment, intestinal adhesion and other postoperative adverse conditions due to the decline of basic organ function and poor tolerance to surgery, as well as the application of general anesthesia drugs.

Acupuncture has a long history of application in the field of analgesia. With the development of acupuncture technology and the emergence of electro-acupuncture (EA) apparatus, its analgesic effect is stronger. At the same time, it has the advantages of convenience, economy, and little body interference (3, 4). It is generally believed that the analgesic and therapeutic effects of EA are superior to those of conventional acupuncture. In recent years, some studies (5, 6) have shown that application of EA anesthesia can strengthen sedation and analgesia, stabilize the internal environment of the body, enhance immunity, and promote body recovery during the perioperative period, and can appropriately reduce the use of local anesthetics. The analgesic effects of EA are mainly manifested as alleviation of diabetic neuropathic pain, postherpetic neuralgia, tactile sensation-induced pain, paclitaxel chemotherapy pain, reduction of cold and heat sensitive pain threshold, and other various types of pain. Based on the above viewpoints, it is of great research significance to explore the effect of EA on tumor resection in elderly patients.

Therefore, this study explores the effect of EA anesthesia combined with general anesthesia in elderly patients with gastrointestinal tumor resection, and analyzes its influence on inflammatory factors, stress state and immune function.

## Methods

### Subjects of Observation

Total of 118 elderly patients who underwent gastrointestinal tumor resection in our hospital from June 2018 to March 2021 were selected and divided into the control group (59 cases) and the observation group (59 cases) according to the random number method. Inclusion criteria: 1. Age ≥60 years old; 2. Have the indications of gastrointestinal tumor resection; 3. Preoperative vital signs were stable; 4. Be tolerated with EA; 5. American Society of Anesthesiologists (ASA) anesthesia classification II–III; 6. The patient or his/her family member have signed informed consent form. Exclusion criteria: 1. Combined with other tumors or important organ dysfunction; 2. There are preoperative cognitive impairment; 3. There are contraindications for general anesthesia. Control group: 32 males and 27 females; Age ranged from 60 to 81 years, with an average of (68.36 ± 5.72) years; The body weight was 52–73 kg, and the average body weight was (62.36 ± 7.95) kg. Observation group: 30 males and 29 females; Age ranged from 60 to 82 years old, and the average age was (69.15 ± 7.99) years old; The body weight was 54–74 kg, and the average body weight was (63.72 ± 9.45) kg. There was no statistical difference between the two groups in general information.

### Treatment Methods

All patients underwent gastrointestinal tumor resection. Relevant signs were monitored, and symptomatic intervention was taken to maintain the stability of vital signs during the operation. There were no significant abnormalities in vital signs such as operation center rate and blood pressure in all patients.

In the control group patients were anesthetized and induced by intravenous midazolam injection (50 µg/kg), phenolphthalein citrate injection (4 µg/kg), propofol emulsion injection (1.5 mg/kg) and vecuronium bromide injection (0.1 mg/kg) before operation. After tracheal intubation, anesthesia was maintained with remifentanil hydrochloride for injection (continuous pumping) and sevoflurane (mask semi-closed inhalation, concentration of 4%, and oxygen flow of 3 L/min).

In the observation group the patients were treated with acupuncture at Baihui (GV20), Hegu (LI 4), Neiguan (PC 6), Shenmen (HT 7) and Zusanli (ST 36) at both sides 20 min before anesthesia induction. After twirling to Deqi, they were connected to the electroacupuncture therapeutic apparatus. The stimulation intensity was adjusted from small to large to the maximum tolerance intensity according to the slow tolerance of the patients, and the density wave was maintained for 20 min. After that, anesthesia induction and anesthesia maintenance were performed, the specific operations were the same as those in the control group.

### Observation Indicators

The anesthesia effects of the two groups were evaluated. Six hours after surgery, the pain was assessed using visual analogue scale scale (VAS) with a total score of 0–10. A higher score indicated more severe pain. The patients’ agitation during the surgery was assessed and divided into 0–3 grades, as shown in **Table 1**. Record the patient’s respiratory normalization time.

**Table 1:** Agitation scoring classification.

0 grade	Quiet cooperation, no restlessness
1 grade	Mild irritability, stimulated surgery patients limbs restlessness, occasional moaning
2 grade	Continuous restlessness and moaning, need to fix the patient’s upper limb
3 grade	Severe agitation, shouting, need to be fixed in patients with upper and lower limbs

Fasting venous blood was collected before surgery, at the end of surgery and 24 h after surgery. The stress state indicators of patients after surgery were detected, including serum cortisol (Cor) by chemiluminescent analyzer, endothelin (ET) by radioimmunoassay, and norepinephrine, (NE) and dopamine (DA) by high-pressure liquid chromatography (HPLC). Enzyme-linked immunosorbent assay was used to detect the serum levels of inflammatory factors 24 h after surgery, including tumor necrosis factor (TNF)-α, interleukin (IL)-6, and IL-1β. The levels of T lymphocyte subsets including $CD_{3}{}^{+}$, $CD_{4}{}^{+}$ and $CD_{4}{}^{+}/CD_{8}{}^{+}$ before and 24 h after surgery were determined by direct immunolabeling with flow cytometry.

Record the number of adverse reactions such as cognitive impairment, nausea, vomiting, intestinal obstruction, intestinal adhesion and so on.

### Statistical Methods

The trial applied EXCEL to collate the relevant data, SPSS 20.0 was applied to calculate the statistical results of the data, and Prism 8.0 was applied to draw the pictures. The measurement data were expressed as mean ± standard deviation (M ± SD), and if the data obeyed normal distribution, the paired t-test was applied to compare the difference between itself before and after treatment within the group, and the t-test of two independent samples was applied to compare the difference between treatment between groups; the count data were expressed as (*n*,%), and the *χ*^2^ test was used for non-rank count data, and the rank sum test was used for rank data. *p *< 0.05 was taken as statistically significant.

## Results

### Comparison of Anesthesia Effects Between Two Groups

**Figure 1:**
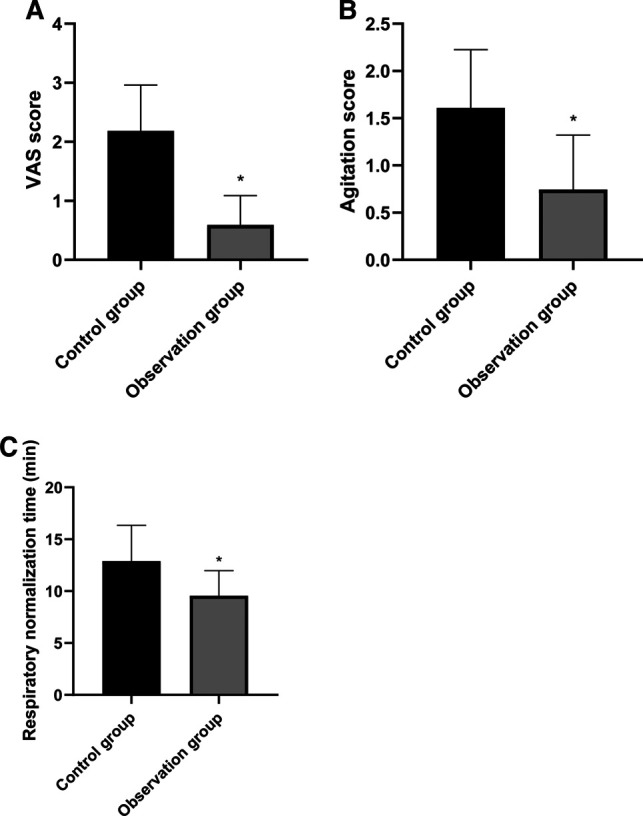
Comparison of anesthesia effects between two groups. Note: Compared with the control group, *p < 0.05.

The VAS score, agitation score and respiratory normalization time in the observation group were lower than those in the control group, and the differences were statistically significant (*p* < 0.05). See **Figure 1** for details**.**

### Comparison of Stress State Index After Surgery Between Two Groups

**Figure 2:**
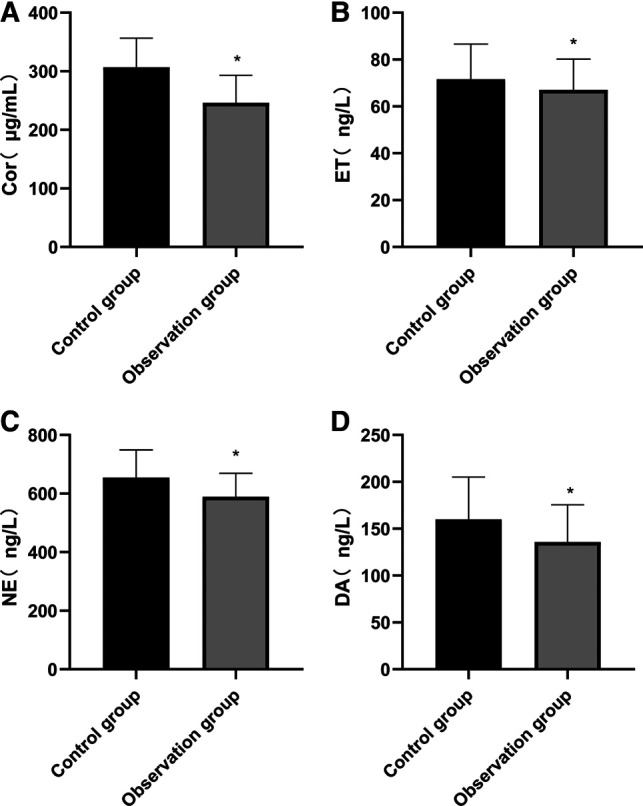
Comparison of stress state index after surgery between two groups. Note: Compared with the control group, *p < 0.05.

After surgery, the levels of serum Cor, ET, NE and DA in the observation group were lower than those in the control group, and the differences were statistically significant (*p* < 0.05). See **Figure 2.**

### Comparison of Postoperative Inflammatory Factor Level Between Two Groups

**Figure 3:**
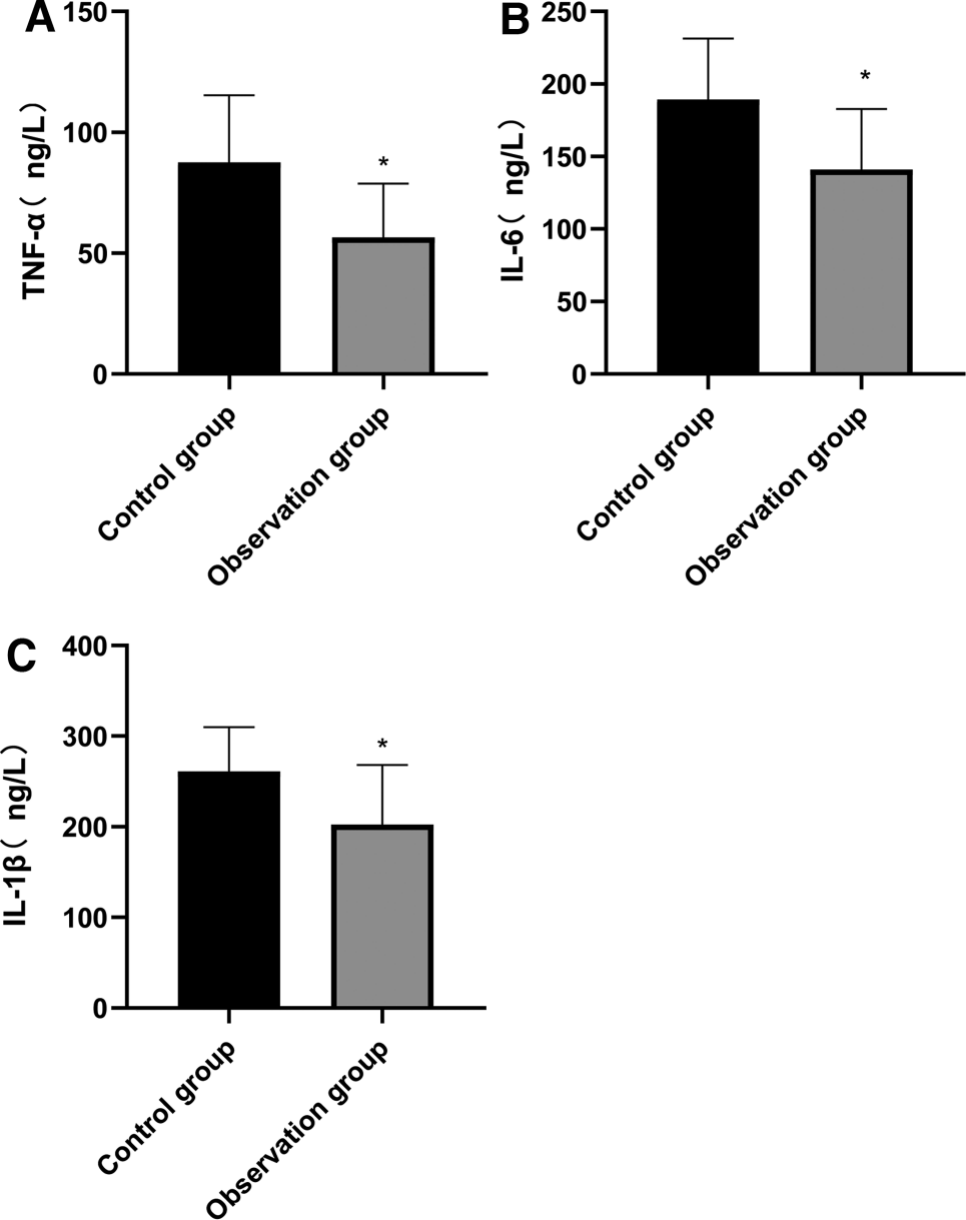
Comparison of postoperative inflammatory factor level between two groups. Note: Compared with the control group, *p < 0.05.

At 24 h after surgery, the levels of serum TNF-α, IL-6 and IL-1β in the observation group were lower than those in the control group, and the differences were statistically significant (*p* < 0.05). See **Figure 3.**

### Comparison of T Lymphocyte Subsets Between Two Groups

**Figure 4:**
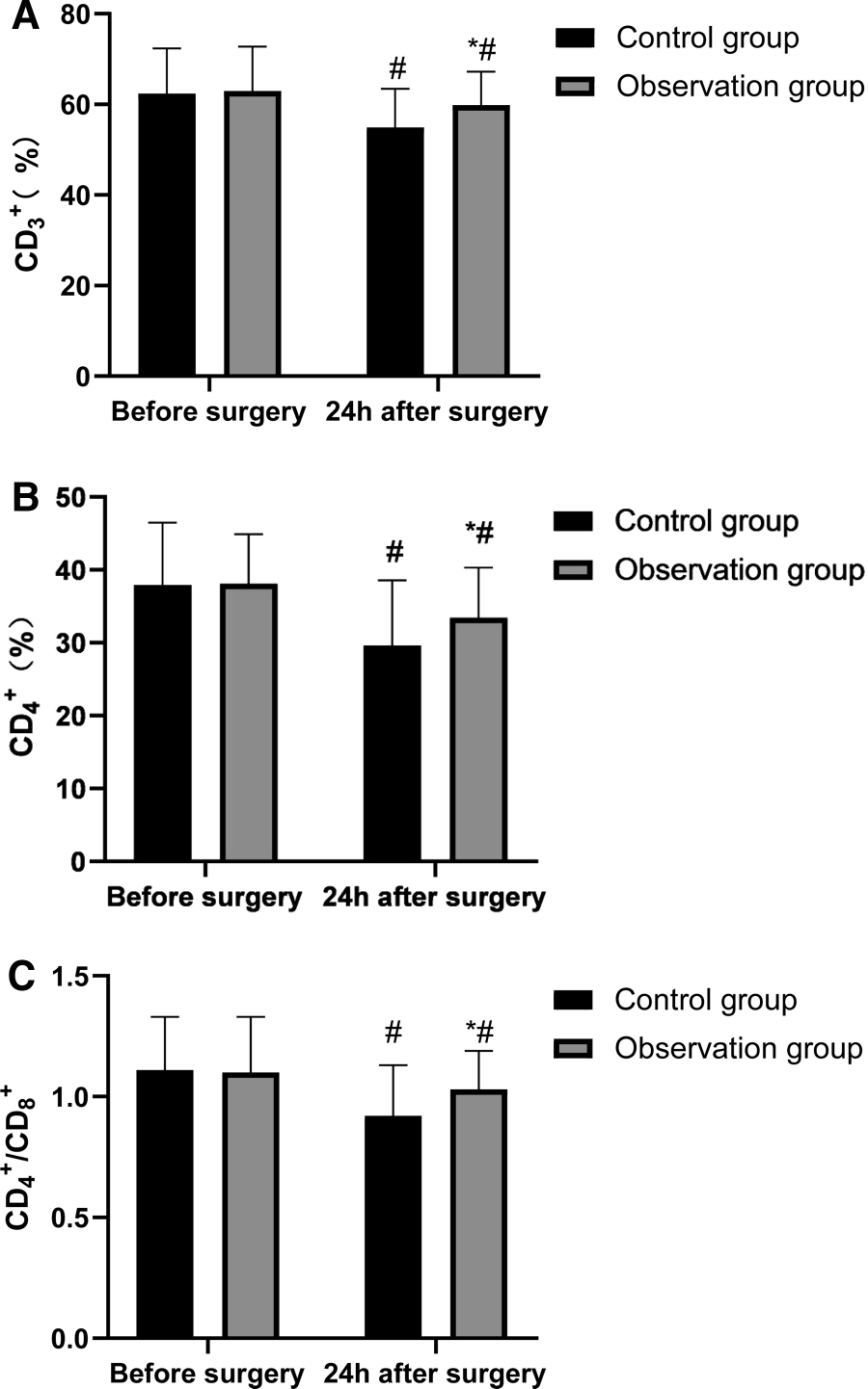
Comparison of T lymphocyte subsets between two groups. Note: Compared with the control group, *p < 0.05; Compared with the same group before surgery, *p < 0.05.

At 24 h after surgery, the levels of $CD_{3}{}^{+}$, $CD_{4}{}^{+}$ and $CD_{4}{}^{+}/CD_{8}{}^{+}$ in the two groups were lower than those before surgery, and the levels of $CD_{3}{}^{+}$, $CD_{4}{}^{+}$ and $CD_{4}{}^{+}/CD_{8}{}^{+}$ in the observation group were higher than those in the control group. The differences were statistically significant (*p* < 0.05). See **Figure 4**.

### Comparison of Adverse Drug Reactions Between the Two Groups During Treatment

During the hospitalization, the total incidence rate of adverse reactions in the observation group after anesthesia was lower than that in the control group, and the difference was statistically significant (*p* < 0.05). See **Table 2**.

**Table 2:** Comparison of adverse drug reactions between the two groups during treatment (*n*,%).

Groups	Cognitive disorder	Nausea and vomiting	Intestinal obstruction	Intestinal adhesion	Total incidence of adverse reactions
Control group (*n* = 59)	5	5	1	1	12 (20.34)
Observation group (*n* = 59)	0	2	0	0	2 (3.40)
*χ*^2^ value					8.104
*p* value					0.004

## Discussion

Elderly patients have low tolerance to narcotic drugs, but general anesthesia is often needed when tumor resection is performed. During this period, severe hemodynamic abnormalities, severe stress response, obvious postoperative anesthesia side effects and high incidence of adverse reactions easily occur (7, 8). Therefore, how to minimize the use of anesthetics and avoid the occurrence of postoperative adverse conditions when ensuring the anesthesia effect is a difficult problem to be solved in general anesthesia surgery for clinical elderly patients (9, 10). Acupuncture analgesia has recently been a hot topic of academic research. Compared with conventional acupuncture, EA is more effective and convenient, so EA is often used to assist anesthesia and analgesia in various surgical procedures. (10, 11). It has been reported that in a variety of local anesthesia surgerys, EA can significantly enhance the perioperative analgesic effect, improve hemodynamic abnormalities, and reduce the incidence of adverse reactions after anesthesia. Regarding the related mechanisms of acupuncture and moxibustion analgesia, it is currently mainly believed that electroacupuncture can regulate the expression and activity of ion channels, regulate the balance of peripheral pro-inflammatory and anti-inflammatory cytokines, inhibit the activation of spinal glial cells, inhibit pain-related signaling pathways at the spinal cord level, and regulate pain-related brain regions and cerebral circuits (12, 13). In our experiment, we selected the five acupoints of Baihui (GV20), Hegu (LI 4), Neiguan (PC 6), Shenmen (HT 7) and Zusanli (ST 36), which are commonly used anesthesia acupoints in acupuncture. At the same time, we have found in the previous clinical practice that the combination of these five acupoints has good analgesic and sedative effects in the treatment of other diseases (14, 15).

When patients undergo surgical trauma, the release of inflammatory mediators will lead to increased nervous system sensitivity, increased pain, significant stress response, and the appearance of agitation (16, 17). The results of this study showed that the patients in the observation group were better than those in the control group in pain severity, agitation and respiratory normalization time after surgery. It was analyzed that EA could stimulate the central nervous system to up-regulate the secretion of morphine-like neurotransmitters with endogenous analgesic effects, such as β -endorphin and at the same time, the signal from acupuncture points could inhibit the transmission of pain signals at nearby spinal stages to the central nervous system, thus increasing the pain threshold of the body (18, 19). In addition, stimulant actions such as tracheal intubation and extubation performed on patients during the surgery will lead to the aggravation of stress response of the body and trigger agitation reaction. However, acupuncture at Hegu (LI 4) exerts analgesic effect and remarkably reduces the sensitivity of the throat to exert a sedative effect (20, 21). Therefore, EA combined with general anesthesia is more effective than general anesthesia alone.

A variety of stimulating operations in tumor resection can lead to the over-activation of the hypothalamic-pituitary-adrenal axis (HPA) and abnormal excitation of the sympathetic center, which in turn causes related reactions of catecholamines, massive release of stress-related neurotransmitters such as serum Cor and ET, and inflammatory factors such as TNF-α and IL-6. However, under the action of long-term high concentration of stress neurotransmitters such as serum Cor and ET and inflammatory factors, patients are vulnerable to accelerated apoptosis of hippocampal neurons and cognitive impairment, which reduces the efficacy of the surgery and has a poor prognosis (22, 23). Stress state can lead to the disorder of endocrine and neurotransmitters in patients, and Cor, ET, NE and DA are the representative indicators. These indicators are usually used to evaluate the stress of the body clinically. The results of this study also showed that the serum levels of Cor, ET, NE, DA, TNF-α, IL-6 and IL-1β in the observation group were lower than those in the control group, indicating that in response to the stress trauma caused by surgery in elderly patients, based on conventional anesthesia, acupuncture could effectively stabilize the internal environment in patients and inhibit the abnormal secretion of neurotransmitters and inflammatory factors, which might be related to the bidirectional regulation of acupuncture on the HPA axis. When the HPA axis was abnormally excited, acupuncture anesthesia could significantly reduce the sympathetic excitability and inhibit catecholamine (24, 25). In addition, stress, inflammatory reaction and tumor can cause the body to be in an immunosuppressed state. Acupuncture at Zusanli (ST 36) and other acupoints has an immunoregulatory effect, and can strengthen cellular and humoral immunity by regulating the number and function of leukocytes, lymphocytes and immunoglobulins (26). In this study, the T lymphocyte subsets in the two groups of patients were lower than those before surgery, while the levels of $CD_{3}{}^{+}$, $CD_{4}{}^{+}$, and $CD_{4}{}^{+}/CD_{8}{}^{+}$ at 24 h after surgery were higher than those in the control group, indicating that acupuncture enhanced the immune function of patients and their immunosuppression due to surgical stress and inflammatory reaction was weak.

Organ function of elderly patients deteriorates. After general anesthesia, central nervous system function is damaged and gastrointestinal motility is decreased due to excessive or intolerance of anesthetics, hemodynamic changes, surgical trauma and other factors, which in turn leads to postoperative cognitive impairment and intestinal adhesion (27). In this study, the adverse reactions of patients in the observation group were only manifested as nausea and vomiting, and no severe adverse reactions such as intestinal obstruction and cognitive impairment was observed. The total incidence of adverse reactions was significantly lower than that in the control group, which might be due to the efficacy of acupuncture in exciting the vgaus nerve, dilating blood vessels, and reducing myocardial oxygen consumption, in addition to inhibiting the excitement of the sympathetic nerve, thereby avoiding excessive disorder of body hemodynamics. In addition, acupuncture can also reduce the penetration of pro-inflammatory factors into the blood-brain barrier, reduce the occurrence of inflammatory reactions in the central nervous system, promote blood perfusion in brain regions, and enhance oxygen supply capacity in the brain, thereby alleviating the cascade reactions of inflammatory injury and protecting cognitive function (28). Therefore, acupuncture combined with general anesthesia is safer, and it is of great significance for the elderly patients who need major surgery.

## Conclusion

In summary, acupuncture anesthesia combined with general anesthesia has good anesthesia effect in elderly patients undergoing gastrointestinal tumor resection, which can stabilize the body environment of patients, alleviate postoperative stress response and inflammatory response, and regulate body immune function. Moreover, it is highly safe and can significantly reduce the occurrence of postoperative adverse reactions.

## Data Availability

The original contributions presented in the study are included in the article/Supplementary Material, further inquiries can be directed to the corresponding author/s.
